# Fatal fulminant hepatitis in a patient taking abiraterone acetate: a case report

**DOI:** 10.3389/fmed.2023.1143244

**Published:** 2023-06-29

**Authors:** Dela Akpokavie, Capucine Gubert, Imene Abdelli, Alix O'Meara Stern, Hervé Zender

**Affiliations:** ^1^Department of Medicine, Service of Internal Medicine, Neuchâtel Hospital Network – La Chaux-de-Fonds, Neuchâtel, Switzerland; ^2^Department of Medicine, Service of Internal Medicine, Neuchâtel Hospital Network – Pourtalès, Neuchâtel, Switzerland; ^3^Department of Oncology, Neuchâtel Hospital Network – La Chaux-de-Fonds, Neuchâtel, Switzerland; ^4^Faculty of Medicine, University of Geneva, Genève, Switzerland

**Keywords:** abiraterone, prostate cancer, hepatotoxicity, fulminant hepatitis, case report

## Abstract

Abiraterone acetate is a steroidal inhibitor of cytochrome P450 17A1 indicated in the treatment of metastatic prostate cancer. This report examines the case of a 66-year-old patient diagnosed with prostate adenocarcinoma that had metastasized to the bones and lymph nodes. Treatment with abiraterone acetate and corticosteroid co-administration as well as LH-RH analog hormone therapy was initiated. Four and a half months later, the patient consulted for deterioration of general condition. Biologically, he developed a fulminant hepatitis of which he eventually died. An infectious or metabolic origin was ruled out. Oncological cause by either disease progression or second neoplastic process was eliminated by means of imaging. Hepatic toxicity was imputed to the treatment with abiraterone acetate. This case suggests that fulminant hepatitis on abiraterone acetate may be underestimated, and underscores the importance of regular monitoring of liver tests on this therapy.

## 1. Introduction

Abiraterone acetate is indicated for the treatment of metastatic castration-resistant (1) and high-risk hormone-induced (2) prostate cancer. It is a semi-synthetic steroidal inhibitor of cytochrome P450 17A1 (CYP17A1), which includes 17 alpha-hydroxylase and 17,20 lyase, key enzymes in the synthesis of steroid hormones (3). Its metabolic effect is to decrease the production of androgens necessary for the growth of hormone-sensitive prostate cancer cells, as well as reducing the production of glucocorticoids. To avoid drug induced adrenal insufficiency, abiraterone acetate is prescribed in combination with prednisone (4).

The most common side effects of abiraterone acetate are fatigue, nausea and/or vomiting, diarrhea and abdominal pain. Other adverse effects are linked to excess mineralocorticoids: fluid retention, hypertension, hypokalemia. Adrenal insufficiency has been described, mainly due to discontinuation of the associated prednisone, or in connection with an increased need for corticosteroids, such as in the context of stress or infection.

Fulminant liver failure is a very rare adverse effect of abiraterone acetate in the treatment of prostate cancer. Only few cases have been described in the literature to date. In this article, we will present a similar case developing fatal fulminant hepatitis.

## 2. Case presentation

### 2.1. Patient information

A 66-year-old patient was diagnosed in March 2020 with Gleason 9 (4 + 5) adenocarcinoma of the prostate, which was metastatic to the bones and lymph nodes. His comorbidities were arterial hypertension and peripheral arterial disease. He was an active smoker with excessive alcohol consumption but with no known underlying liver disease. Treatment consisted of bicalutamide for 1 month in order to avoid a “flare up” syndrome, associated thereafter with a treatment of leuprorelin (LHRH), that was later supplemented with abiraterone acetate (1 g/day) plus prednisone (5 mg/day) starting April 2020. Treatment adherence was good, and the patient was regularly followed.

On August 23, 2020 the patient presented to the emergency department because of a three-day decline in general condition associated with fatigue, lack of appetite, and nausea without vomiting. He had had no food intake for 48 h and very little water intake, consequently describing anuria for 24 h. The patient was also taking paracetamol (4 g/d). The last consultation was 20 days earlier, with liver tests being within normal limits. The patient was not known to have any underlying liver disease.

### 2.2. Clinical findings at admission

On physical examination, the patient had a low blood pressure at 86/46 mmHg, which corrected rapidly after intravenous hydration, hypothermia at 33°C, and adequate saturation on 2 liters/min of oxygen. He was cachectic, icteric with mottled skin discoloration of the extremities. He experienced epigastric pain on palpation, without Murphy's sign. The neurological status was normal except for drowsiness (Glasgow coma scale of 14/15).

In the laboratory, we observed severe capillary hypoglycemia at 1.6 mmol/l, a significant increase in aminotransferases, as well as cholestasis: ASAT 14′492 U/l, ALAT 6′126 U/l, alkaline phosphatase 338 U/l, gamma-glutamyl transpeptidase 633 U/l, total bilirubin 96 μmol/l. The rest of the work-up showed a KDIGO stage 3 acute kidney injury with creatinine at 205 μmol/l, a severe thrombocytopenia at 16 G/l and an INR prolongation at 3.89. Severe metabolic acidosis was also found with a pH of 6.99, a PaCO_2_ of 2.7 kPa, bicarbonates of 5 mmol/l and hyperlactatemia of 13.7 mmol/l.

### 2.3. Diagnostic assessment

The etiologies of the acute liver damage were investigated. The ceruloplasmin assay was within the norm. Ferritin was 5,260 μg/l, transferrin saturation was 93%, serum iron 30.2 μmol/l and transferrin 1.3 g/l. The paracetamol level was not elevated at 105 μmol/l, with a last intake 8 h earlier (5).

Hepatitis serology came back negative for viral hepatitis A and C and indicated previous hepatitis E infection as well as previous vaccination for hepatitis B. EBV, CMV and HSV 1 serologies show acquired immunity, HIV and HSV 2 serologies were negative. Blood cultures, urine cultures and sputum cultures all came back sterile.

Abdominal ultrasound showed a normal liver, without cirrhosis or biliary dilatation. The Doppler demonstrated patency of the portal vein and the suprahepatic veins with physiological circulatory flow.

The cervico-thoraco-abdominal CT scan without contrast medium revealed a right apical pulmonary nodule and an increase in size and number of bone metastases. Imaging also demonstrated bilateral pleural effusions.

### 2.4. Therapeutic intervention

In the emergency room, oxygen therapy at 2 l/min was started for 24 h, because of pulmonary overload, probably in the setting of associated acute kidney injury. Intravenous hydration was used to rapidly correct hypotension. Blood glucose was also corrected intravenously.

A potential bacterial infection was treated empirically with broad-spectrum antibiotics (piperacillin-tazobactam), after sampling. In addition, a transient increase in corticosteroid therapy with intravenous hydrocortisone was initially undertaken in the context of a suspected adrenal insufficiency component in a patient on long-term prednisone. Basal cortisol levels returned to normal, allowing resumption of the usual prednisone dosage.

The patient was admitted to intensive care and abiraterone acetate treatment was withheld. Intravenous N-acetylcysteine was given for the management of fulminant hepatitis (6). Osmotic laxative therapy was also introduced to treat the hepatic encephalopathy. In the absence of diuresis and without response to high-dose diuretics, daily dialysis sessions were undertaken. Antibiotic therapy was stopped after 5 days, in the absence of any clear evidence of infection.

### 2.5. Follow-up and outcomes

**Figure 1:**
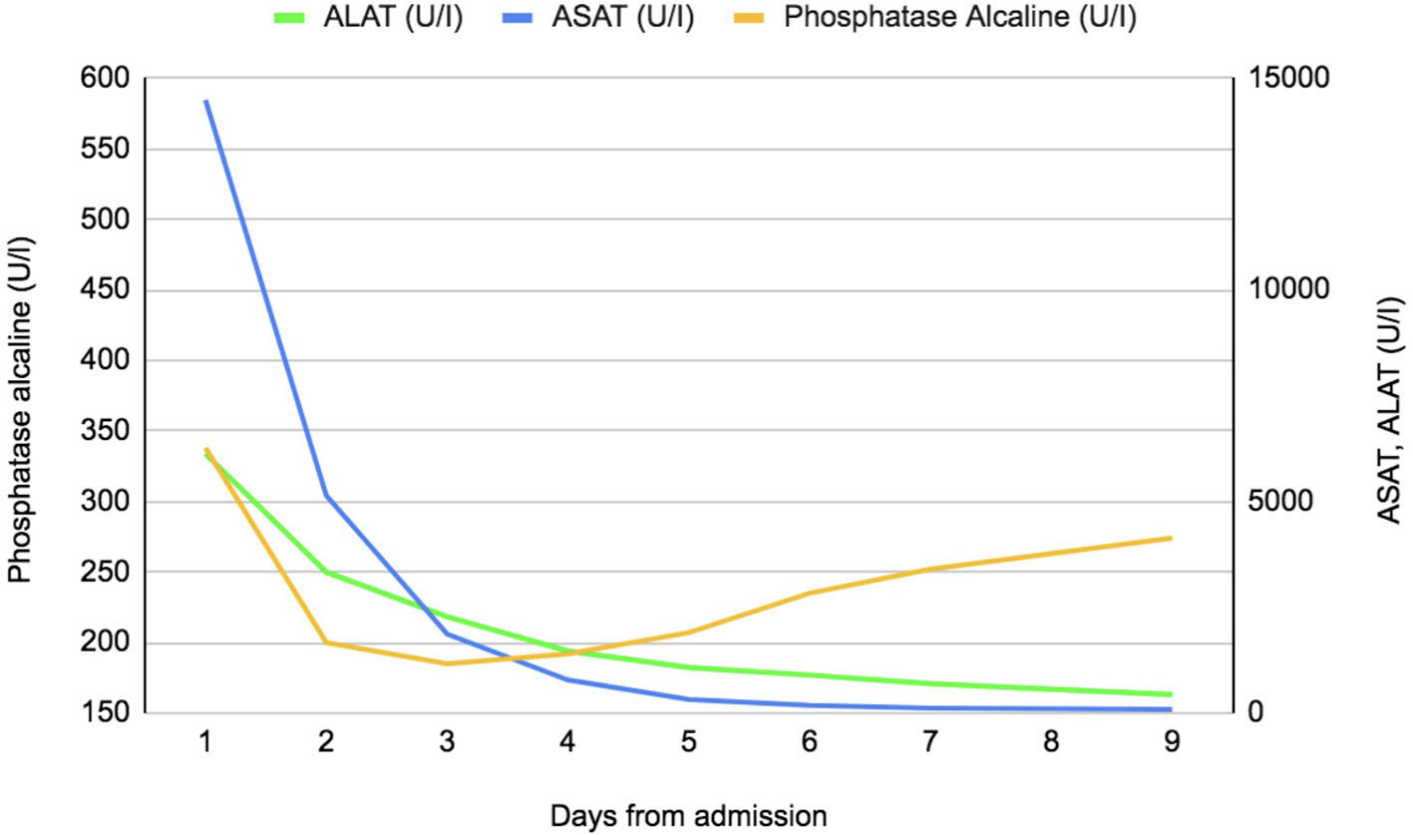
Evolution of daily liver tests since admission.

The patient remained hemodynamically stable during his stay. Liver enzymes decreased rapidly (Figure 1), but the patient did not recover his renal function and anuria persisted requiring continued daily dialysis sessions. We considered this to be secondary KDIGO stage 3 acute kidney injury, in the context of an AKI-type hepatorenal syndrome (7).

Finally, the patient's neurological state deteriorated with drowsiness and confusion (Glasgow Coma Score 13/15) in the context of encephalopathy. The latter is probably of mixed origin on the concomitant hepatic and renal insufficiency.

Given the rapid deterioration of his neurological condition, the absence of a therapeutic, strategy the patient's comorbidities and in agreement with his family intensive care measures were ceased. The patient died on September 1, 2020 of multi-organ failure secondary to acute hepatocellular insufficiency, 10 days after his admission. The hypothesis of fulminant hepatic failure due to abiraterone acetate was retained.

## 3. Discussion

We are faced with a picture of fulminant hepatitis according to the definition of Polson and Lee (8): absence of underlying liver disease, coagulopathy with INR ≥ 1.5, encephalopathy and an evolution of <26 weeks. The clinical manifestations of fulminant hepatitis were abrupt: the neurological state deteriorated rapidly as it can occur in the context of hepatic encephalopathy, progressing to cerebral oedema and coma in a few days-weeks (9).

In terms of etiology, significant cytolysis may indicate ischemic or toxic liver injury in over 90% of cases of acute liver injury (10). With regard to other causes of liver damage, the workup allowed us to exclude the common causes of acute liver failure.

Therehere was no laboratory evidence of acute viral hepatitis. The ceruloplasmin assay was within the norm, ruling out Wilson's disease. A hemochromatosis was possible in view of a high ferritin and a transferrin saturation level > 45%, but this alone does not explain the appearance of fulminant hepatitis (a mutation of the HFE gene was not sought). Liver ultrasound excluded a primary or metastatic neoplastic origin, biliary tract involvement, vascular occlusion or hepatic steatosis.

An autoimmune work-up was not performed but this was unlikely due to the sudden onset of the biological liver damage. An ischemic cause due to the initial hypotension was not formally excluded, but was very unlikely in view of the rapidly reversible hypotension after filling (11).

Factors that speak in favor of abiraterone acetate toxicity are the absence of confounding risk factors–such as massive alcohol abuse, excessive paracetamol use, alternative medicine, antibiotic or concomitant chemotherapy use. Improvement in liver tests after discontinuation of this treatment may be consistent with a drug-induced etiology. Finally, the absence of an obvious alternative etiology should be noted.

As far as limiting factors are concerned, the premature death of the patient does not allow a detailed follow-up of the clinical case. The absence of liver biopsy and autopsy is another limiting factor as these examinations could have confirmed our hypothesis or, less likely, showed another etiology.

Liver toxicity of abiraterone acetate has been described to varying degrees. Increases in aminotransferases occur in up to 13% of patients treated with abiraterone acetate compared to 1% to 8% on placebo. These abnormalities are usually transient and asymptomatic (12).

In the LATITUDE trial, a phase 3 study with 1,199 randomized patients of whom 597 received abiraterone acetate, there was an increase in ALT of grade 1–2 in 11% of cases, grade 3 in 5% of cases and grade 4 in <1% of cases. For AST, there was an increase of grade 1–2 in 11% of cases, grade 3 in 4% of cases and grade 4 in <1% of cases (13).

In the COU-AA-301 trial, with 1,195 patients randomized, 797 of whom received abiraterone acetate, an early grade 4 elevation in aminotransferases led to a protocol amendment specifying more frequent monitoring of liver function during the first 12 weeks of treatment. Overall, abnormalities in liver function enzymes occurred with similar frequency in the abiraterone acetate and placebo groups, including all grades of changes in liver function tests (10% and 8%, respectively), grade 3 or 4 changes in liver function tests (3.5% and 3.0%), grade 3 or 4 elevations in AST (1.4% and 1.6%), grade 3 or 4 elevations in ALT (1.0% and 1.1%), and grade 4 elevations in aminotransferases (0.3% and 0.5%) (14).

Liver enzymes disturbances are also described in a French retrospective study of 25 patients with metastatic prostate cancer undergoing treatment from 2009 to 2017 (15). This study shows an increase in transaminases (ASAT and ALAT concomitantly) occurring mainly after 2 months of abiraterone acetate treatment. The condition is typically asymptomatic and in the majority of cases normalizes after a median of 2 months, either spontaneously or after reduction or discontinuation of treatment. According to the Common Terminology Criteria for Adverse Events (CTCAE) of the National Cancer Institute of NIH, grade 1, 2 mainly, up to 3 (moderate to severe requiring hospitalization) side effects are described following abiraterone acetate treatment (16). Similarly, the Naranjo (adverse drug reaction probability scale) score shows a causality of 4 to 5 (possible to probable) when linking abiraterone acetate treatment to the adverse effect on liver function (17).

Several cases of fatal fulminant hepatitis have been described after the drug was marketed. One case was published in the US in 2017 in a Chinese-American patient who developed fulminant hepatitis 8 weeks after the introduction of the drug, with death within 12 days of hospital admission (18). In Japan, seven cases were reported between 2014 and 2017, with three deaths, three remissions and no clear data on the outcome of the last case. Among them, one case has been published, having presented fulminant hepatitis within 1 month of the introduction of abiraterone acetate (19). Finally, Scailteux et al. listed nine hospitalizations for hepatotoxicities (acute and fulminant hepatitis) reported in the French national database between 2013 and 2018, among which seven deaths occurred (20).

The pathophysiology of abiraterone acetate-induced liver damage is still unknown. An interaction with CYP3A4 and 2D6 leading to the production of toxicants or immunological intermediates has been suggested (12).

According to the drug label, monitoring liver function is recommended when starting the treatment, with adaptation or discontinuation of the treatment in case of hepatic impairment (21).

## 4. Conclusion

Fulminant liver failure following abiraterone acetate treatment is a rare side effect in the literature and may be more common in a post-marketing setting (22). This case underscores the importance of close monitoring of liver function at the start of treatment. This is particularly important as this drug has become a cornerstone in the management of metastatic prostate cancer, significantly increasing survival (23). Knowing how to recognize the full range of these adverse effects, particularly on liver function, therefore seems to be crucial in medical practice.

## Data availability statement

The original contributions presented in the study are included in the article/supplementary material, further inquiries can be directed to the corresponding author.

## Ethics statement

Written informed consent was obtained from the individual(s) or from the individuals' next of kin for the publication of any potentially identifiable images or data included in this article.

## Author contributions

DA and CG: performed the literature review, wrote the manuscript, contributed to its revision, read, and approved the submitted version. IA: wrote sections of the manuscript. AS: contributed to the manuscript's revision, read, and approved the submitted version. HZ: contributed to the manuscript's conception, its revision, read, and approved the submitted version. All authors contributed to the article and approved the submitted version.
